# The pyrazole derivative of usnic acid inhibits the proliferation of pancreatic cancer cells in vitro and in vivo

**DOI:** 10.1186/s12935-023-03054-x

**Published:** 2023-09-24

**Authors:** Mariola Gimła, Agnieszka Pyrczak-Felczykowska, Marcelina Malinowska, Aleksandra Hać, Magdalena Narajczyk, Irena Bylińska, Tristan A. Reekie, Anna Herman-Antosiewicz

**Affiliations:** 1 Department of Medical Biology and Genetics, Faculty of Biology, University of Gdańsk, Wita Stwosza 59, 80-308 Gdańsk, Poland; 2 Department of Physiology, Medical University of Gdańsk, 80-211 Gdańsk, Poland; 3 Electron Microscopy Section, Faculty of Biology, University of Gdańsk, 80-308 Gdańsk, Poland; 4 Department of Biomedical Chemistry, Faculty of Chemistry, University of Gdańsk, 80-308 Gdańsk, Poland; 5 School of Science, University of New South Wales Canberra, Australian Capital Territory, Canberra, 2600 Australia

**Keywords:** Cancer, Usnic acid, Endoplasmic reticulum stress, Cell death, Lichens, Vacuolization

## Abstract

**Background:**

Pancreatic cancer is one of the leading causes of cancer death in Western societies. Its late diagnosis and resistance to chemotherapies result in a high mortality rate; thus, the development of more effective therapies for the treatment of pancreatic cancer is strongly warranted. Usnic acid (UA) is a secondary metabolite of lichens that shows modest antiproliferative activity toward cancer cells. Recently, we reported the synthesis of a UA pyrazole derivative, named **5**, which was more active than the parent compound toward cervical cancer cells. Here, its anticancer potential has been evaluated in detail in other cancer cells, particularly pancreatic cancer cells.

**Methods:**

The impact of UA and derivative **5** on cell viability, morphology, cell cycle, and death was assessed using the MTT test, electron microscopy, flow cytometry, and immunoblotting, respectively. The calcium ions level was detected fluorometrically. In vivo*,* the anticancer activity of **5** was evaluated in a murine xenograft model.

**Results:**

Derivative **5** inhibited the viability of different cancer cells. Noncancerous cells were less sensitive. It induced the release of calcium ions from the endoplasmic reticulum (ER) and ER stress, which was manifested by cell vacuolization. It was accompanied by G0/G1 cell cycle arrest and cell death of pancreatic cancer cells. When applied to nude mice with xenografted pancreatic cancer cells, **5** inhibited tumor growth, with no signs of kidney or liver toxicity.

**Conclusions:**

UA derivative **5** is superior to UA inhibiting the growth and proliferation of pancreatic cancer cells. ER stress exaggeration is a mechanism underlying the activity of derivative **5**.

**Supplementary Information:**

The online version contains supplementary material available at 10.1186/s12935-023-03054-x.

## Introduction

Pancreatic cancer is the seventh leading cause of cancer deaths in both sexes. In 2020, it accounted for almost as many deaths (446.000) as cases (496.000). Incidence and mortality rates are fourfold and fivefold higher in highly developed countries, and the highest incidence rates are detected in Europe, North America, Australia and New Zealand [1]. Survival rates for pancreatic cancer remain low, despite improvements in overall 5-year survival from < 5% in the 1990s to 9% in the USA and Europe in 2019. Low survival rates are, in part, due to the advanced stage at diagnosis in most cases, with only ~ 20% of patients with early-stage, surgically resectable disease [2]. The majority of cancers in the pancreas (> 90%) are pancreatic ductal adenocarcinomas [3].

Therapies used to treat pancreatic cancer (surgery, radiotherapy and conventional chemotherapies) have only modest effects on survival length. For example, only 5.4% of patients are sensitive to gemcitabine, a first-line chemotherapy agent. Thus, effective novel therapies are urgently needed to treat this disease.

Usnic acid (C_18_H_16_O_7_) [2,6-diacetyl-7,9-dihydroxy-8,9b-dimethyldibenzofuran-1,3(2*H*,9b*H*)-dione; UA] is a secondary metabolite found in lichens and has been shown to possess a broad spectrum of biological activities, including antiproliferative, anti-metastatic and anti-angiogenic activities, that might protect against cancer development or progression (reviewed in [4–8]). UA has been shown to display cytotoxicity against a wide panel of murine and human cancer cells in vitro, albeit at rather high concentrations (reviewed in [6]). For instance, the IC_50_ of ( +)-UA in lung squamous cell carcinoma (H520 and Calu-1) was 32–34 µM after 48 h of treatment [9], in colon (HCT116 and HT-29) and ovarian cancer cells (A2780) after 72 h of treatment was 100–157 µM and 76 µM, respectively [10], and the IC_50_ of (-)-UA in glioblastoma cells (T89G and A-172) was 38 µM (13 µg/mL) or 91 µM (31.5 µg/mL) after 48 h of treatment [11]. More recently, UA activity against gastric cancer cells was evaluated, and IC_50_ values after 24 h of treatment were calculated as approximately 237 μM for BGC823 cells and 619 μM for SGC7901 cells [12].

Importantly, quite controversial results related to UA safety have been reported. When used as a supplement to induce human weight loss, UA revealed unwanted hepatotoxic effects. Depending on the supplement used, the daily intake of UA could reach 300—1350 mg and was used from a few weeks to 3 months [13, 14]. In primary cultured murine hepatocytes, 5 µM UA induced necrosis in 98% of cells within 16 h, and it was associated with inhibition and uncoupling of the electron transport chain in mitochondria, leading to a reduction in ATP levels in hepatocytes [15]. Intraperitoneal injections of UA suspension at a dose of 15 mg/kg/day for 15 days in male Swiss mice caused hepatic dysfunction, as revealed by a high level of serum transaminase and histological observation of necrotic areas in livers [16]. Therefore, research efforts concentrate on the modification of the UA structure to obtain derivatives with higher potency against cancer cells and lower side effects toward healthy cells.

Recently, we reported the synthesis of UA derivatives that are more cytotoxic toward cancer cells than the parent compound [17, 18]. Moreover, the isoxazole derivative of UA induced massive vacuolization of MCF-7 breast cancer cells, which resulted from endoplasmic reticulum (ER) stress and led to paraptosis-like cell death [18, 19].

In this study, we tested a new pyrazole UA derivative, named **5** ((*R*)-8-acetyl-5,7-dihydroxy-3,4a,6-trimethyl-1,4a-dihydro-4*H*-benzofuro[3,2-*f*]indazol-4-one), that is superior to recently described **2** and **3a** toward HeLa cancer cells (the IC_50_ values determined after 24 h of treatment were approximately four- and ninefold lower, respectively) [17]. We show that similar to isoxazole derivative **2**, pyrazole derivative **5** induced ER stress in breast MCF-7 cells and also in pancreatic cancer cells. As pancreatic cells exhibit high secretory functions and therefore are characterized by highly developed ER, exacerbation of ER stress has been proposed as a promising target for pancreatic cancer therapy [20, 21]. Thus, we further concentrated on the activity of compound **5** toward pancreatic cancer cells in in vitro and in vivo models.

## Materials and methods

### Reagents used in the study

Procedures for the synthesis of UA pyrazole derivative **5** have been described in [17]. Fetal bovine serum, DMEM, penicillin/streptomycin antibiotic mixture and Matrigel were purchased from Corning (USA). ( +)-UA, DMSO, and thiazolyl blue tetrazolium bromide (MTT) were purchased from Sigma‒Aldrich (St. Louis, MO, USA).

Antibodies against GRP78/BiP, and anti-rabbit, anti-mouse, and anti-β-actin antibodies conjugated with horseradish peroxidase were purchased from Sigma‒Aldrich. Antibodies against IRE1α and GADD153/CHOP were from Santa Cruz Biotechnology (Santa Cruz, CA, USA), and an antibody against PARP was purchased from Cell Signaling Technology (Danvers, MA, USA). The inhibitors: 2-aminoethoxydiphenylborane (2-APB); 1,2-bis(2-aminophenoxy)ethane-*N*,*N*,*N*',*N*'-tetraacetic acid (BAPTA) were purchased from Sigma‒Aldrich.

### Cell culture conditions

The human breast adenocarcinoma cell line MCF-7 was obtained from CLS Cell Lines Service GmbH (Eppelheim, Germany), human dermal fibroblasts HDFa were obtained from Thermo Fisher Scientific (Product Line Cascade Biologics™), and the human pancreatic cancer cell lines MIA PaCa-2 and PANC-1, were provided by Dr. I. Inkielewicz-Stępniak from the Medical University of Gdansk, Poland. All cell lines were tested for mycoplasma contamination before their use.

Monolayer cultures of MCF-7 cells were maintained in RPMI 1640; HDFa, Mia PaCa-2 and PANC-1 cells were maintained in DMEM (4 mM L-glutamine and 4500 mg/L of glucose). Basic media were supplemented with 10% (v/v) fetal bovine serum and a 1% penicillin‒streptomycin mixture. Each cell line was maintained at 37 °C in a humidified atmosphere with 5% CO_2_.

### Cell viability assay

Cell viability was determined by the MTT method. Cells were seeded at a density of 4 × 10^3^ (for 24 h tests) or 2 × 10^3^ (for 48 h tests) per well of a 96-well plate and allowed to attach overnight. The medium was replaced with fresh medium supplemented with desired concentrations of **5** for 24 h or 48 h. In some experiments, cells were pretreated with 2-APB (30 μM) or BAPTA (10 μM) for one hour. Before the end of treatment, 25 µl of MTT solution (4 mg/mL) was added to each well. After 3 h of incubation, the medium was removed, and formazan crystals were dissolved in 100 µl of DMSO. Absorbance was measured at 570 nm (with a reference wavelength of 660 nm) in a Victor^3^ microplate reader. Data were obtained from at least three independent experiments performed in triplicate. IC_50_ values were calculated using GraphPad Prism software. The selectivity index was calculated as the IC_50_ value in the normal cell line divided by the IC_50_ value in the cancer cell line.

### Measurement of Ca^2+^ level

Cells were seeded at a density of 2 × 10^4^ per well in a 96-well plate and allowed to attach overnight. The medium was replaced with fresh medium supplemented with desired concentrations of **5** for 6 h (MCF-7) and 12 h (Mia PaCa-2, PANC-1). In some experiments, cells were pretreated with 2-APB (30 μM) or BAPTA (10 μM). The Ca^2+^ level was evaluated using a Fluo-4 Direct Assay Kit (Invitrogen) according to the manufacturer’s instructions.

### Cell cycle and cell death determination

The effect of the investigated compounds on cell cycle distribution and cell death was determined by Muse™ Cell Analyzer (Millipore). Cells were seeded in 6-well plates at a density of 2 × 10^5^ per well. After 24 h, the cells were treated with 1 or 5 µg/mL derivative **5**, UA, or an equivalent amount of DMSO. After 24 h (cell cycle) or 48 h (cell death), both medium and trypsinized cells were collected, centrifuged for 10 min at 300 × g, stained using the Muse™ Cell Cycle Kit or Muse ™ Annexin-V & Dead Cell Assay Kit and counted by flow cytometry.

### Transmission electron microscopy (TEM)

Transmission electron microscopy was performed essentially as described previously [18]. Briefly, cells (2 × 10^5^) were plated in 12-well plates and allowed to attach overnight. Next, the cells were treated with either DMSO (control), 1 or 5 μg/mL **5** for 24 or 48 h at 37 °C. For TEM, cells were fixed in ice-cold 2.5% electron microscopy grade glutaraldehyde (Polysciences) in PBS (pH 7.4). The samples were rinsed with PBS, postfixed in 1% osmium tetroxide with 0.1% potassium ferricyanide, dehydrated through a graded series of ethanol washes (30–100%), and embedded in Epon (Fluka). Semithin (300 nm) sections were cut using an RMC Power Tome XL ultramicrotome, stained with 0.5% toluidine blue and examined under a light microscope. Ultrathin sections (65 nm) were cut using a Leica UC7 ultramicrotome, stained with Uranyless (Delta Microscopies) and Reynold’s lead citrate (Delta Microscopies), and examined on a Tecnai G2 Spirit BioTWIN transmission electron microscope at 120 kV.

### Immunoblotting

Cells were treated with **5** or UA (1 or 5 µg/mL for 6 or 24 h) and lysed using a solution containing 50 mM Tris (pH 7.5), 1% Triton X-100, 150 mM NaCl, 0.5 mM EDTA, and protease and phosphatase inhibitor cocktails (Roche Diagnostics). The lysates were cleared by centrifugation. Proteins were separated by SDS‒PAGE and transferred onto a PVDF membrane. The membrane was blocked with 5% nonfat dry milk in phosphate-buffered saline and incubated with the desired primary antibody overnight at 4 °C. The membrane was then treated with the appropriate secondary antibody for 1 h at room temperature. Immunoreactive bands were detected with an enhanced chemiluminescence reagent (Thermo Scientific). Blots were stripped and reprobed with anti-β-actin antibodies to normalize for differences in protein loading. Each protein was detected three times in independently prepared lysates. Densitometry analysis was carried out using Quantity One 1-D Analysis software (Bio-Rad).

### Animal studies

The experiments on mice were conducted at the Tri-City Academic Laboratory Animal Centre (Gdańsk). The animal protocol was approved by the Local Ethics Committee for Animal Experimentation in Bydgoszcz (permit No. 20/2019). Animal experimentation was performed in accordance with EU directive 2010/63/EU. Female BALB/c-Nude mice (CAnN. Cg-Foxn1nu/Crl, 5 weeks old) were purchased from AnimaLab (Poznań, Poland). Animals were housed in IVC cages under 12 h light and dark cycles with food and water ad libitum. Animals were adapted to the experimental conditions for one week before the start of the experiment. To generate tumor xenografts 5 × 10^6^ Mia PaCa-2 cells in Matrigel were injected subcutaneously into the flanks of each mouse. When the tumor volume reached approximately 80 mm^3^, the mice were randomly divided into groups (n = 9 in each group). Animals were treated for 4 weeks, 3 times a week by oral gavage with corn oil (control group) or **5** suspended in corn oil (400 mg/kg). Tumor growth and body weight were recorded every 2–3 days. At the end of the experiment, the mice were sacrificed, and the tumors, livers and kidneys were excised, measured, and stored for further analysis.

### Histopathology

Dissected tumors, livers and kidneys were fixed with 4% paraformaldehyde in PBS and paraffin-embedded. Five-micrometer-thick sections were mounted on Superfrost Plus adhesive slides (Thermo Scientific, USA). All samples were stained with hematoxylin and eosin (H&E, Eosin Y, Harris Hematoxylin Shandon, Thermo Scientific) to determine the tissue structure and degree of vacuolization. Slides were mounted with DPX (Fluka, Switzerland). All comparative sections were performed at the same time using identical conditions. Images were taken using an Olympus light microscope IX51 with a CCD camera and CellSens Software.

### Statistical analysis

All data are shown as the means ± standard errors (SE) of at least three independent experiments. The significance of differences between the control and treated cells in viability tests was analyzed with ANOVA and Dunnett’s or Sidak’s multiple comparison post hoc tests using GraphPad Prism (version 8). Differences were considered significant at *P* < 0.05.

## Results

### UA pyrazole derivative is active against cancer cells of different origins

**Fig. 1.**
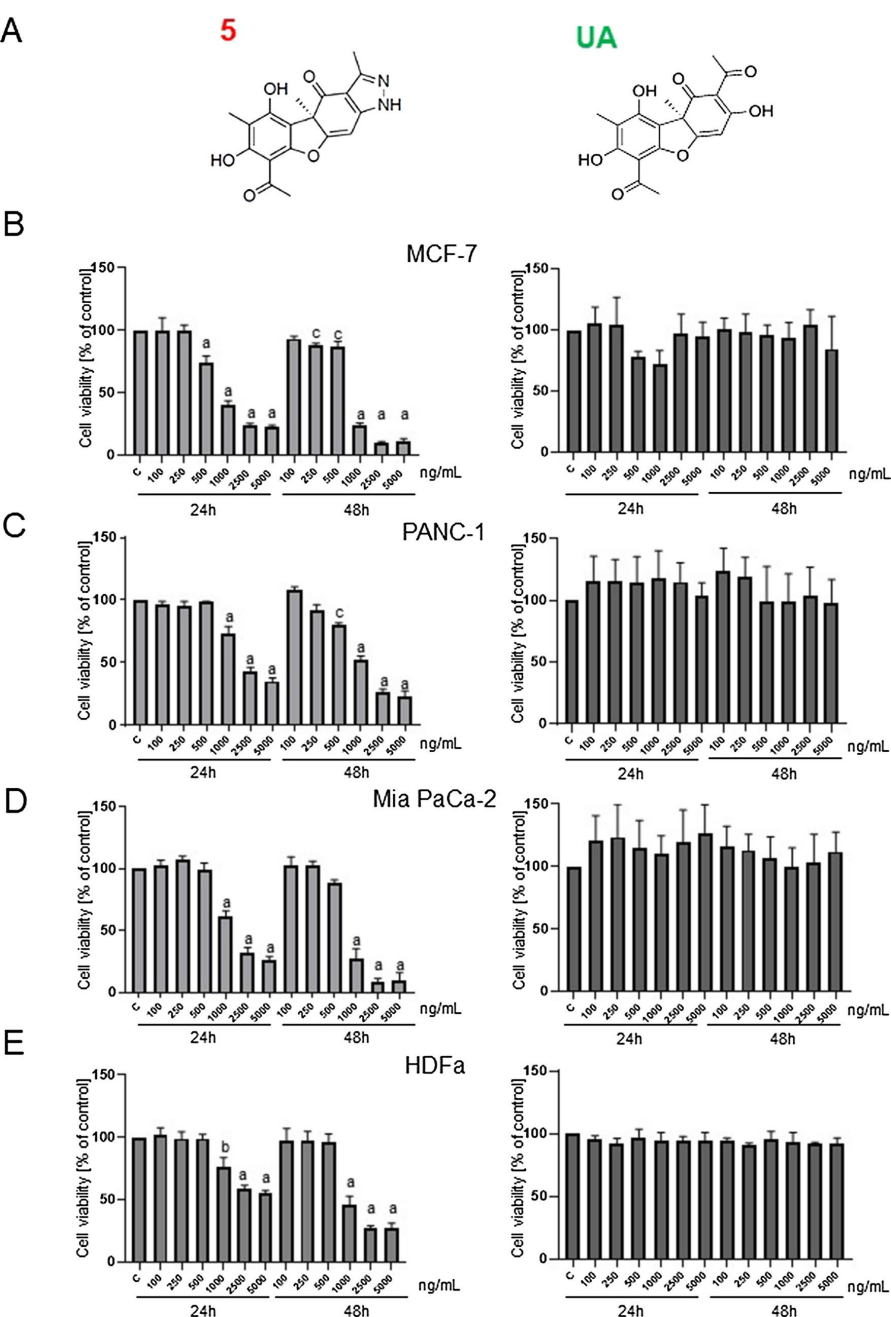
**5,** but not UA, decreases the viability of cancer cells, and noncancerous cells are less sensitive. **A** Chemical structures of compound **5** and UA. **B**-**E** Viability of MCF-7, PANC-1, Mia PACa-2 (cancer cells) and HDFa (noncancerous cells). Viability was measured using an MTT test after 24 or 48 h of treatment with **5** or UA at the indicated concentrations. Statistical significance between control and **5**-treated cells was determined by ANOVA followed by Dunnett’s post hoc test: a—*P* < 0.0001, b—*P* < 0.001, c—*P* < 0.05. Experiments were performed 3–6 times in triplicate

The UA pyrazole derivative, (*R*)-8-acetyl-5,7-dihydroxy-3,4a,6-trimethyl-1,4a-dihydro-4*H*-benzofuro[3,2-*f*]indazol-4-one, named **5** (Fig. 1A), was synthesized as described in [17] and revealed potent activity against HeLa cervical cancer cells [17]. Here, its anticancer potential was evaluated against a panel of human cancer cells derived from different organs as well as normal cells using the MTT viability assay. As Table 1 shows, derivative **5** more efficiently decreased the viability of cancer cells than healthy fibroblasts (selectivity index ranged from 1.8 for PANC-1 to 7.2 for HeLa cells after 24 h of treatment). It was more potent than previously investigated derivatives **2** (isoxazole) or **3** (pyrazole) against MCF-7 (the IC_50_ determined after 24 h of treatment was threefold lower than that for derivative **2**) and HeLa cells (the IC_50_ values were approximately fourfold- and ninefold lower than for **2** and **3**, respectively) [17, 18]. Mia PaCa-2 and PANC-1 pancreatic cancer cells appeared to be less sensitive to derivative **5** than MCF-7 cells; however, the IC_50_ values after 48 h of treatment were still close to 1 µg/mL, and **5** reduced the viability of cells in a dose- and time-dependent manner, contrary to parental UA (Fig. 1B-D). Skin fibroblasts were more resistant to derivative **5** than cancer cells (Fig. 1E).

**Table 1:** Sensitivity (IC_50,_ µg/mL) of different cancer and noncancerous cell lines to usnic acid derivative **5** after 24 or 48 h of drug treatment

Origin	Cell line	IC_50_ (24 h)	IC_50_ (48 h)
breast	MCF-7	1.10	0.82
cervix	HeLa	0.66	0.29
lung	A549	0.71	0.46
liver	HepG2	0.80	0.36
colon	HCT15	2.09	1.38
pancreas	PANC-1	2.71	1.35
pancreas	Mia PaCa-2	1.96	0.90
skin fibroblasts	HDFa	4.78	1.68

Each value is the mean of at least three experiments

**Fig. 2:**
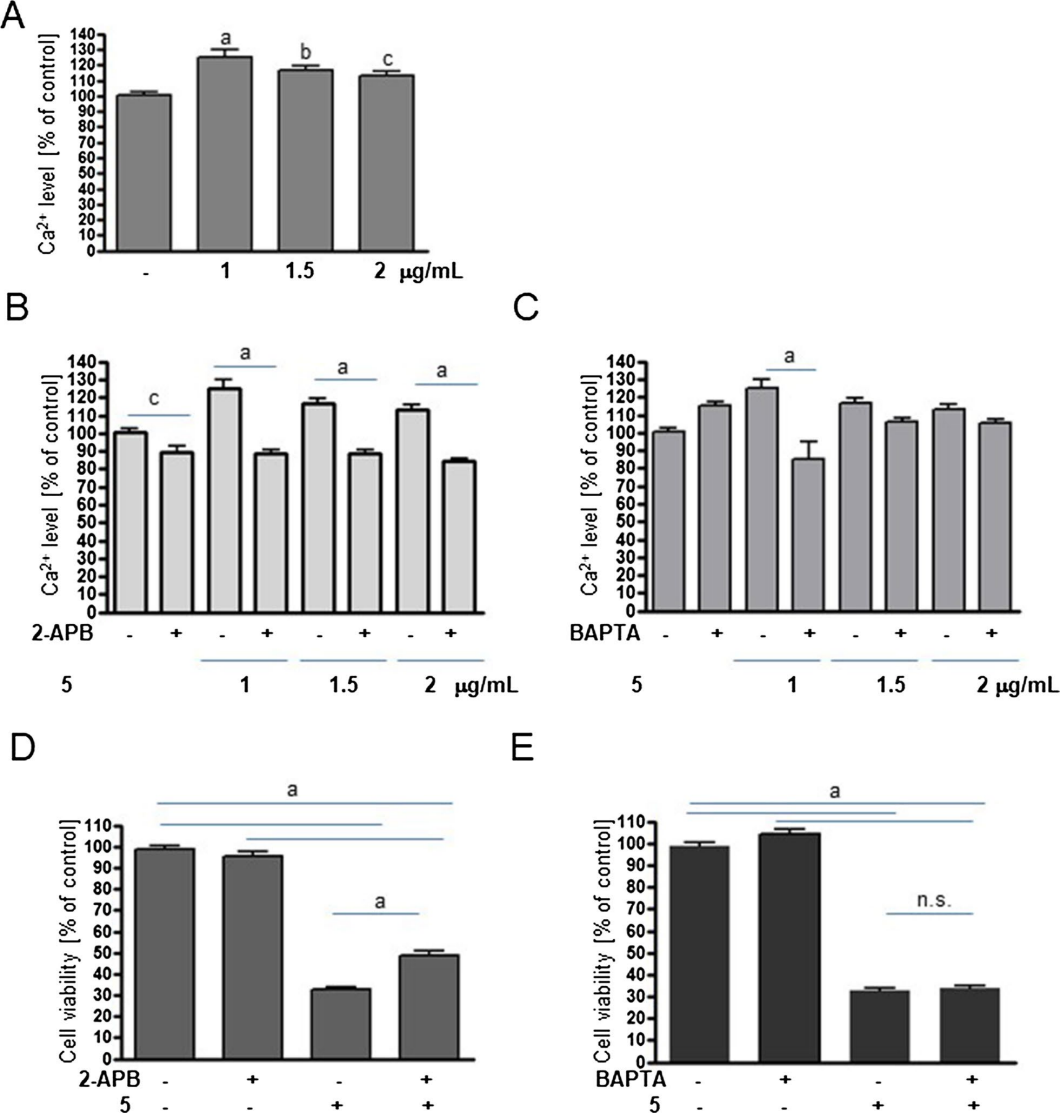
UA derivative **5** elevates cytosolic Ca^2+^ levels in breast cancer cells. **A** Relative level of Ca^2+^ in MCF-7 cells treated with vehicle (DMSO, 100%) or **5** at the indicated concentrations for 6 h. **B**-**C** Effect of 2-APB (30 µM, **B**) or 2- BAPTA (10 µM, **C**) 1-h pretreatment on Ca^2+^ levels in MCF-7 cells treated or not with compound **5**. **D**-**E**: Effect of 2-APB (**D**) or BAPTA (**E**) pretreatment on the viability of MCF-7 cells treated or not with **5** for 24 h. The data are shown as the mean ± SE (n = 7–11). Statistical significance was determined by ANOVA followed by Dunnett’s (**A**) or Sidak’s (**B-E**) post hoc tests: a—*P* < 0.0001, b—*P* < 0.001, c—*P* < 0.05, n.s.—not significant

Recently, it has been shown that UA isoxazole derivative** 2** elevates cytosolic Ca^2+^ levels in MCF-7 cells, leading to ER stress [19]. To elucidate whether derivative **5** acts in a similar way, MCF-7 cells were treated with **5**, and cytosolic Ca^2+^ levels were evaluated. As shown in Fig. 2A, derivative **5** increased the level of Ca^2+^. This effect resulted from Ca^2+^ release from the ER as an inhibitor of IP3 receptors, 2-APB, blocked this process, in contrast to BAPTA, a nonpermeable extracellular calcium chelator, which did not (Fig. 2B-C). Moreover, inhibition of Ca^2+^ leakage from the ER by 2-APB partially protected against a **5**-induced drop in MCF-7 viability, while BAPTA had no effect (Fig. 2D-E).

**Fig. 3:**
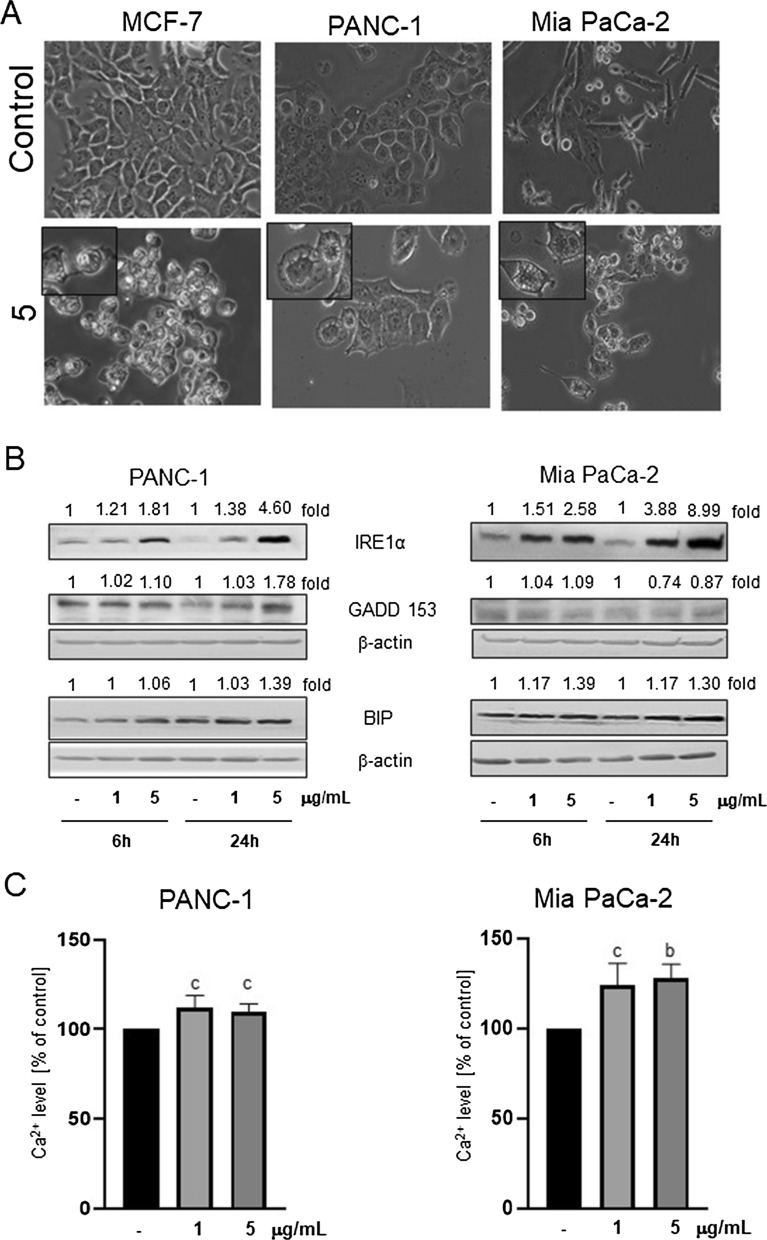
Derivative **5** induces vacuolization and ER stress. **A**: Morphology of MCF-7, PANC-1 and Mia PaCa-2 cells examined under light microscopy (magnification 200x). Cells were treated with DMSO (control) or 1 µg/mL of derivative **5** for 48 h. Enlarged sections of samples treated with **5** are shown in the insets. **B**: Immunoblots for ER stress markers, BIP, IRE1, and GADD153 in pancreatic cancer cells treated with DMSO or **5** (1 or 5 µg/mL) for 6 or 24 h. The blots were stripped and reprobed with the anti-β-actin antibody to ensure equal protein loading. Densitometric scanning data after correction for loading control (mean of 3 repetitions) are above the immunoreactive bands. **C**: Relative level of Ca.^2+^ in pancreatic cancer cells (PANC-1 and Mia PaCa-2) treated with vehicle (DMSO, 100%) or **5** at the indicated concentrations for 12 h. The data are shown as the mean ± SE (n = 7–11). Statistical significance was determined by ANOVA followed by Dunnett’s post hoc test: **b**—*P* < 0.001, **c**—*P* < 0.05

It was reported that some cancers, such as pancreatic cancer, are characterized by constant ER stress, and its elevation might be an efficient way to eradicate them [21, 22]; thus, Mia PaCa-2 and PANC-1 pancreatic cancer cells were used to elucidate whether derivative **5** impacts their ER homeostasis. As shown in Fig. 3A, **5** induced vacuolization of pancreatic cancer cells, similar to MCF-7 cells. Markers of ER stress, such as BIP, IRE1α, GADD153, were elevated, as revealed by immunoblotting (Fig. 3B). Moreover, derivative **5** increased Ca^2+^ levels in the cytoplasm of pancreatic cancer cells (Fig. 3C), which was inhibited by 2-APB but not by BAPTA (Additional file 1: Fig. S1). Emptyfication of Ca^2+^ stores might be the cause of ER stress induction, as in the case of MCF-7 cells.

**Fig. 4:**
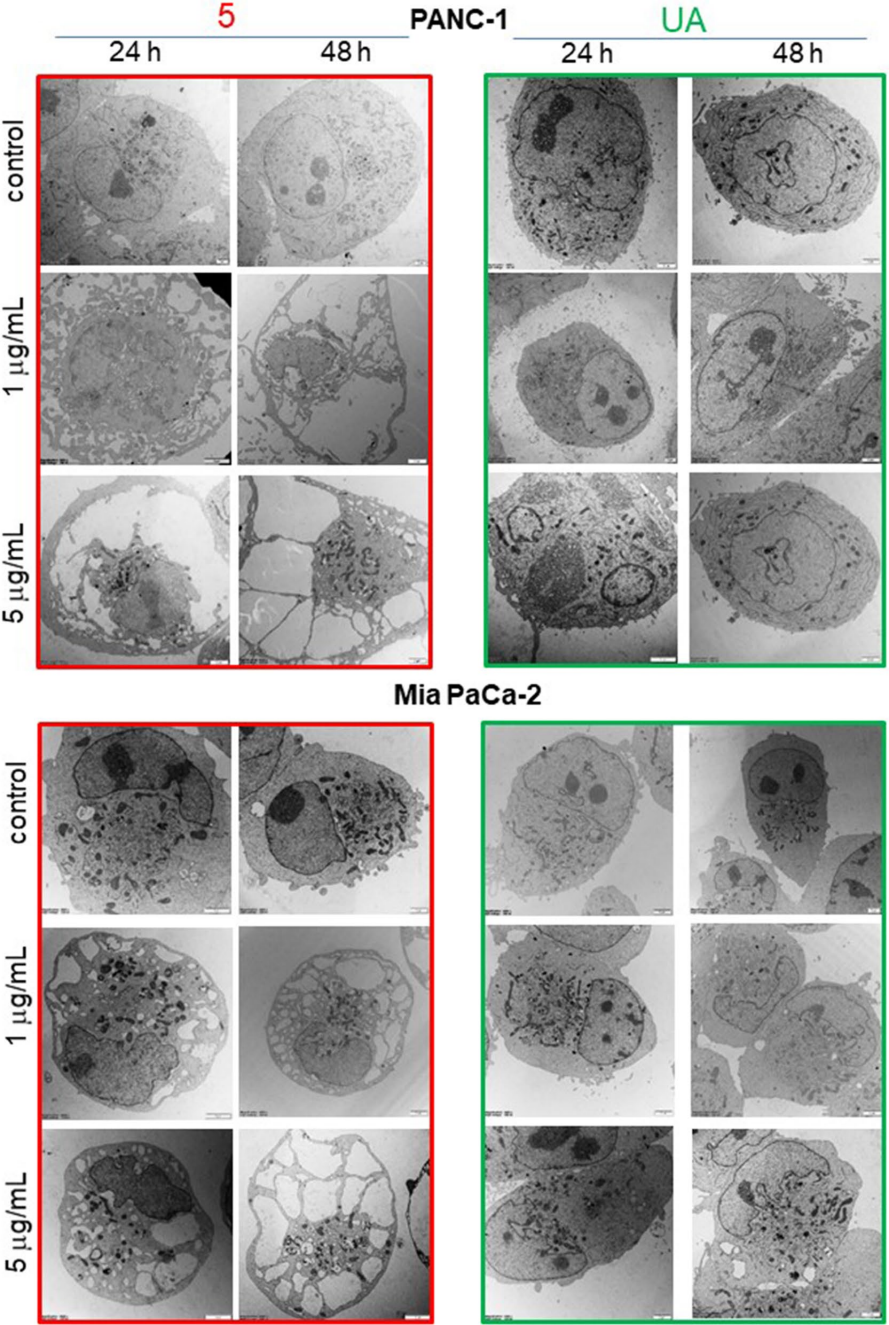
Derivative **5** but not parental UA induces massive vacuolization of pancreatic cancer cells. Morphology of PANC-1 and Mia PaCa-2 cells examined under TEM. Cells were treated with DMSO (control), **5** or UA at 1 or 5 µg/mL for 24 or 48 h. Representative pictures of cells at magnifications of 2900x, 4800 × or 6800 × are shown

A more detailed investigation of the ultrastructure of pancreatic cancer cells revealed dose- and time-dependent vacuolization upon treatment with **5,** and no such features were observed in cells treated with UA at the same concentrations (Fig. 4). Analysis of electron microscopy images indicated that large vacuoles that appear in pancreatic cancer cells are of ER origin, which supports ER stress induction. In the case of PANC-1, cells with damaged plasmalemma have also been found, which is a feature of necrotic death. In contrast, UA used at similar concentrations had minimal effect on pancreatic cancer cell morphology (Fig. 4).

### UA derivative 5 inhibits cell cycle progression and induces cell death in pancreatic cancer cells

To further elucidate the mechanisms of the antiproliferative activity of derivative **5** in pancreatic cancer cells, its effect on the cell cycle and cell death was investigated. As shown in Table 2, treatment with derivative **5** for 24 h increased the percentage of G0/G1 cells in a dose-dependent manner, and at the same time, the number of cells in the S and G2/M phases decreased. UA at the same concentrations had minimal effects on the cell cycle.

**Table 2:** Derivative **5** blocks pancreatic cancer cell cycle progression in G0/G1 phase in a time-dependent manner

Name	Concentration [µg/mL]	Cell cycle phase [%]		
		G0/G1	S	G2/M
PANC-1				
Control		43.8 ± 1.3	20.4 ± 0.9	34.5 ± 1.7
( +) usnic acid	1	44.3 ± 2.6^ns^	20.5 ± 0.8^ns^	33.8 ± 3.4^ns^
	5	42.7 ± 1.1^ns^	18.9 ± 1.2^ns^	36.3 ± 1.9^ns^
**5**	1	47.8 ± 2.3^ns^	18.8 ± 2.1^ns^	31.9 ± 1.2^ ns^
	5	61.9 ± 4.3^a^	8.1 ± 2.2^b^	28.4 ± 2.3^ns^
Mia PaCa-2				
Control		42.3 ± 1.1	29.9 ± 1.7	33.5 ± 1.2
( +) usnic acid	1 5	39.8 ± 1.4^ns^ 41.2 ± 2.7^ns^	24.3 ± 3.6^ns^ 24.9 ± 3.6^ ns^	33.2 ± 2.4^ns^ 31.4 ± 2.1^ns^
**5**	1	48.0 ± 1.5^ns^	19.2 ± 1.7^ns^	29.5 ± 2.6^ns^
	5	54.5 ± 0.8^a^	15.7 ± 0.9^ns^	26.9 ± 1.7^c^

Cells were treated with 1 or 5 µ g/mL UA or derivative **5** for 24 h. Each value is the mean (± SE) of three experiments. Statistical significance was determined with ANOVA and Sidak’s multiple comparison test and a – *P* < 0.0001, b – *P* < 0.001, c – *P* < 0.05, ns – not significant

**Fig. 5:**
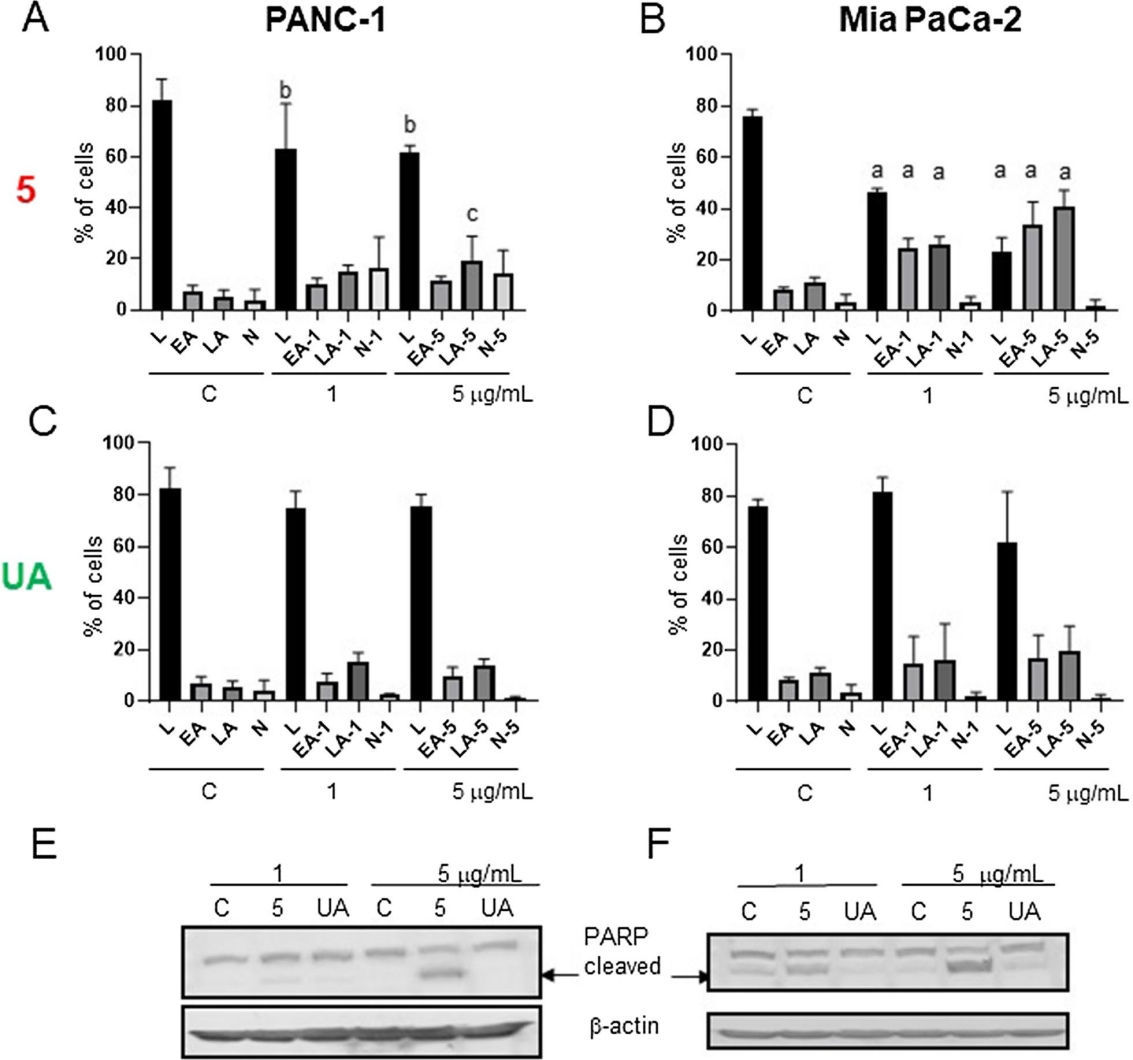
Derivative **5** induces the death of pancreatic cancer cells. **A**-**D**: PANC-1 (**A**, **C**) or Mia PaCa-2 (**B**, **D**) cells were treated with DMSO (control, **C**), **5** (upper graphs—**A**, **B**) or UA (lower graphs—**C**, **D**) at 1 or 5 µg/mL for 48 h. The amounts of live (L), early apoptotic (EA), late apoptotic (LA), and necrotic (N) cells were determined by flow cytometry after staining with Muse™ Annexin V and Dead Cell Kit. The results are presented as the mean ± SE of 3–6 independent experiments. Statistical significance between the respective control and **5**-treated fractions was determined with ANOVA and Sidak’s post hoc test and is marked with a (*P* < 0.0001), b (*P* < 0.001), and c (*P* < 0.05). **E**: PANC-1 and **F**: Mia PaCa-2 immunoblots for caspase-cleaved PARP. The blots were stripped and reprobed with an anti-β -actin antibody to ensure equal protein loading

Analysis of cancer cell death using the detection of phosphatidylserine by Annexin V and membrane permeabilization by the accumulation of 7-aminoactinomycin D (7-AAD) dye indicated that derivative **5** decreased the number of viable cells and increased the number of apoptotic cells, particularly in the case of the Mia PaCa-2 cell line: derivative **5** at a 5 µg/mL concentration elevated the total fraction of apoptotic cells from 20% to over 70% after 48 h of treatment (Fig. 5B). In the case of PANC-1, viable cells dropped from 80 to 60%, and there were 30% and 10% apoptotic and necrotic cells, respectively, upon treatment with 5 µg/mL derivative **5** (Fig. 5A). UA was less effective in the induction of cell death than derivative **5** (Fig. 5C-D). Immunoblotting for caspase-cleaved PARP confirmed that **5** is more active than UA in apoptosis induction, particularly in Mia PaCa-2 cells (Fig. 5E-F).

### Orally administered derivative 5 retards Mia PaCa-2 xenograft growth in mice

**Fig. 6:**
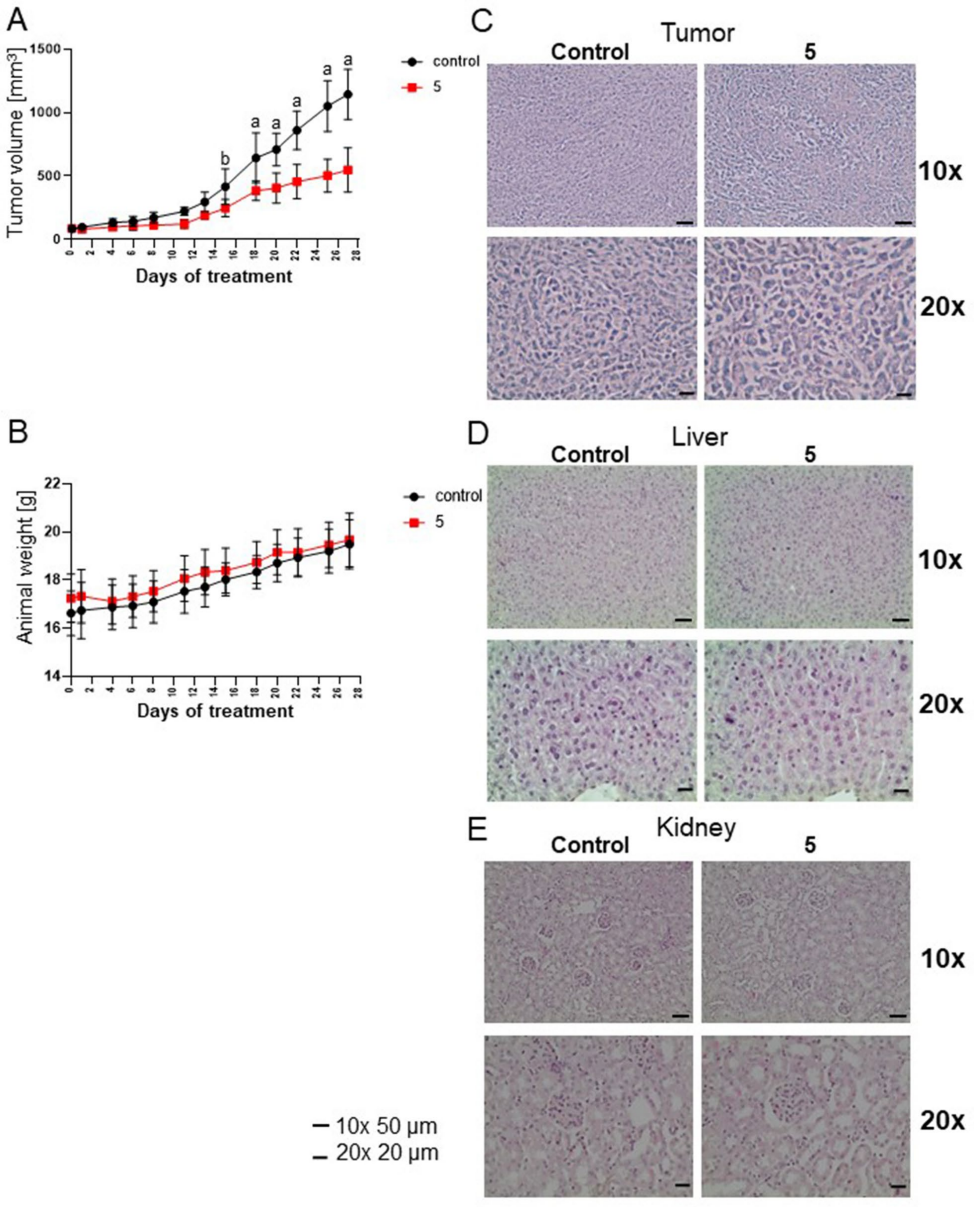
Derivative **5** inhibits the growth of Mia PaCa-2 cell xenografts in nude mice. Effect of **5** (400 mg/mL) or vehicle (corn oil) treatment on the tumor volumes (**A**) and body weights of animals (n = 9) (**B**). Statistical significance between the control and **5**-treated groups was determined by ANOVA followed by Sidak’s post hoc test: a—*P* < 0.0001, b—*P* < 0.001. **C**-**E**: Histology of tumor (**C**), liver (**D**) or kidney (**E**) sections in control and **5**-treated mice. Images were taken under a 10 × or 20 × objective, and representative results are shown. Tissue sections were fixed, embedded in paraffin, sectioned, and processed for H & E staining

The results presented in this work indicate that **5** is more potent than UA as an antiproliferative agent against cancer cells, including pancreatic cancer cells. To elucidate whether **5** is active in vivo as well, we tested it in murine models. The acute toxicology tests based on the oral administration of **5** to laboratory strain BALB/c mice allowed for the determination of the Maximum Tolerated Dose as 400 mg/kg (data not shown). Next, the effect of **5** on the growth of Mia PaCa-2 xenografts in nude mice was tested. As shown in Fig. 6A, orally administered **5** inhibited tumor growth, which was evident after 6 doses. The tested derivative did not affect body mass (Fig. 6B).

Histopathological analysis showed that derivative **5** changed the structure of the tumor, which might be connected with cancer cell death induction (Fig. 6C). Vacuolization of tumor cells can be noticed in **5**-treated animals. Importantly, such changes were not observed in the livers and kidneys of either control or **5**-treated animals (Fig. 6D-E).

## Discussion

This work shows that the new pyrazole UA derivative **5** induces cell cycle arrest and death of pancreatic cancer cells and is connected with ER stress induction. The tested compound is much more active than parental UA, which, when used at the same concentrations, has almost no effect on cancer cells.

There are only a few reports investigating the activity of UA or its derivatives in pancreatic cancer cells. Previously, the activity of ( +)-UA from *Cladonia arbuscula* and ( −)-UA from *Alectoria ochroleuca* was tested in T47D breast and Capan-2 pancreatic cancer cells. Both enantiomers revealed similar anti-proliferative effects against tested cell lines (the IC_50_ was 4.2 μg/mL and 4.0 μg/mL for ( +) and ( −)-usnic acid against T47D, and 5.3 μg/mL and 5.0 μg/mL against Capan-2, respectively) measured by ^3^H thymidine incorporation into DNA. UA reduced cancer cell size and at 10 µg/mL, induced G0/G1 cell cycle arrest and mitochondrial membrane depolarization. It also caused necrosis but only in Capan-2 pancreatic cancer cells exposed for 48 h to 5 or 10 µg/mL UA [23]. Later, the same authors confirmed that 10 µg﻿/mL UA caused necrosis but not apoptosis in tested cell lines [24]. Modifications of the UA structure revealed that some derivatives show enhanced activity compared to the parent compound. UA enamine with an imidazole substituent (2e (R, *E*)-6-acetyl-2-[1-(3-(1*H*-imidazol-1-yl) propylamino)ethylidene]-7,9-dihydroxy-8,9b-dimethyldibenzofuran-1,3(2*H*,9b*H*)-dione) and UA pyrazole (4a, (R)-8-acetyl-5,7-dihydroxy-3,4a,6-trimethyl-1-phenyl-1*H*-benzofuro[3,2-f]indazol-4(4a*H*)-one) revealed potent antiproliferative activity against cervix (HeLa), breast (MDA-MB-231), lung (A549) and pancreas (Mia PaCa-2) cancer cells with IC_50_ values of approximately 4–6 and 6–7.5 µM, respectively, in an SRB viability assay [25]. A more detailed examination in HeLa cells showed that both derivatives used at a 10 µM concentration for 24 h destabilized microtubules, which led to G2/M block in the cell cycle [25].

Here, we showed that pyrazole derivative **5** inhibited pancreatic cancer cell viability, inducing cell cycle arrest in the G0/G1 phase and cell death. Mia PaCa-2 cells were slightly more sensitive to **5,** which at 1 or 5 µg/mL concentration induced mainly apoptosis after 48 h of treatment (51 and 74% apoptotic cells, respectively, compared to 20% in controls). In PANC-1 cells, derivative **5** at 1 or 5 µg/mL moderately induced apoptosis (24 and 27 vs. 14%), but necrotic cells were also detected in flow cytometry experiments (13 and 11 vs. 5%) and TEM images. PANC-1 cell cycle arrest was higher than that in Mia PaCa-2 cells treated for 24 h with 5 µg/mL derivative **5** (62% and 55%, respectively, compared to 43% in controls). Such differences in response to derivative **5** might be related to the genetic background of these cell lines. Nevertheless, **5** induced massive vacuolization in both pancreatic cancer cell lines, similar to breast cancer cells. Vacuolization resulted from ER stress accompanied by the release of ER-stored Ca^2+^ to the cytosol, which was observed in breast and pancreatic cancer cells.

Solid tumors often suffer from hypoxia, oxidative stress and deprivation of nutrients, including glucose. All these processes are well-known inducers of ER stress. This accumulation of misfolded proteins in the lumen of the ER triggers the unfolded protein response (UPR) which restores homeostasis in this organelle. It relies on changes in gene expression to produce enzymes engaged in protein folding or degradation and in the inhibition of global translation to reduce translational load. This is accompanied by the expansion of ER size [26, 27]. In the majority of cases, the UPR plays an adaptive pro-survival role. However, persistent and unresolved ER stress leads to cell death [28]. Pancreatic cells exhibit a high level of synthesis and secretion of hormones and digestive enzymes; therefore, they possess a highly developed ER, which makes them especially sensitive to ER stress-induced apoptosis [20, 29]. Highly developed ER is also observed in pancreatic cancer cells. Moreover, pancreatic tumors show a high basal level of ER stress [30]. Therefore, ER targeting is regarded as a promising approach to the therapy of pancreatic cancer [21, 22].

## Conclusions

The results presented in this work indicate that the antiproliferative activity of UA derivative **5** relies on ER stress induction in cancer cells, including pancreatic cancer cells, both growing in vitro and in an animal model. Importantly, the treatment of animals with **5** neither had adverse effects nor affected the morphology of healthy organs. These features make derivative **5** a promising candidate for future research on its use for the treatment of pancreatic cancer patients.

### Supplementary Information

**Additional file 1.** **Figure S1.** Elevation of cytosolic Ca^2+^ levels in pancreatic cancer cells results from its release from ER. The relative level of Ca^2+^ in PANC-1 (**A**) and Mia PaCa-2 (**B**) cells treated with vehicle (DMSO, 100%), **5** at indicated concentrations with or without inhibitor of IP3 receptors (2-APB at 30 μM, left panels) or extracellular calcium chelator (BAPTA, 10 μM, right panels) for 12 h. The data are shown as the mean ± SE (n=6-9). Statistical significance was determined by ANOVA followed by Sidak’s post-hoc tests: a - P < 0.0001, b - P < 0.001, c - P < 0.05, n.s. - not significant.

## Data Availability

The data supporting the findings of this study are available from the corresponding author upon reasonable request.

## Abbreviations

2-APB: Aminoethoxydiphenylborane
BAPTA: 1,2-Bis(2-aminophenoxy)ethane-*N*,*N*,*N*',*N*'-tetraacetic acid
BiP: Binding immunoglobin protein
ER: Endoplasmic reticulum
IRE1α: Inositol requiring enzyme 1 alpha
DMSO: Dimethyl sulfoxide
GADD153: Growth arrest DNA damage 153
PARP: Poly(ADP-ribose) polymerase
PBS: Phosphate-buffered saline
SE: Standard error
UA: ( +)-Usnic acid
UPR: Unfolded protein response
vs.: Versus
