# Supplementary material for: The pyrazole derivative of usnic acid inhibits the proliferation of pancreatic cancer cells in vitro and in vivo
Source: Cancer Cell Int. 2023 Sep 24;23:210. doi: 10.1186/s12935-023-03054-x (PMC10518105; doi:10.1186/s12935-023-03054-x)
Supplement: Supplementary file 1 — Additional file 1. Figure S1. Elevation of cytosolic Ca2+ levels in pancreatic cancer cells results from its release from ER. The relative level of Ca2+ in PANC-1 (A) and Mia PaCa-2 (B) cells treated with vehicle (DMSO, 100%), 5 at indicated concentrations with or without inhibitor of IP3 receptors (2-APB at 30 μM, left panels) or extracellular calcium chelator (BAPTA, 10 μM, right panels) for 12 h. The data are shown as the mean ± SE (n=6-9). Statistical significance was determined by ANOVA followed by Sidak’s post-hoc tests: a - P < 0.0001, b - P < 0.001, c - P < 0.05, n.s. - not significant. [file 12935_2023_3054_MOESM1_ESM.docx]

**Fig. S1**

**
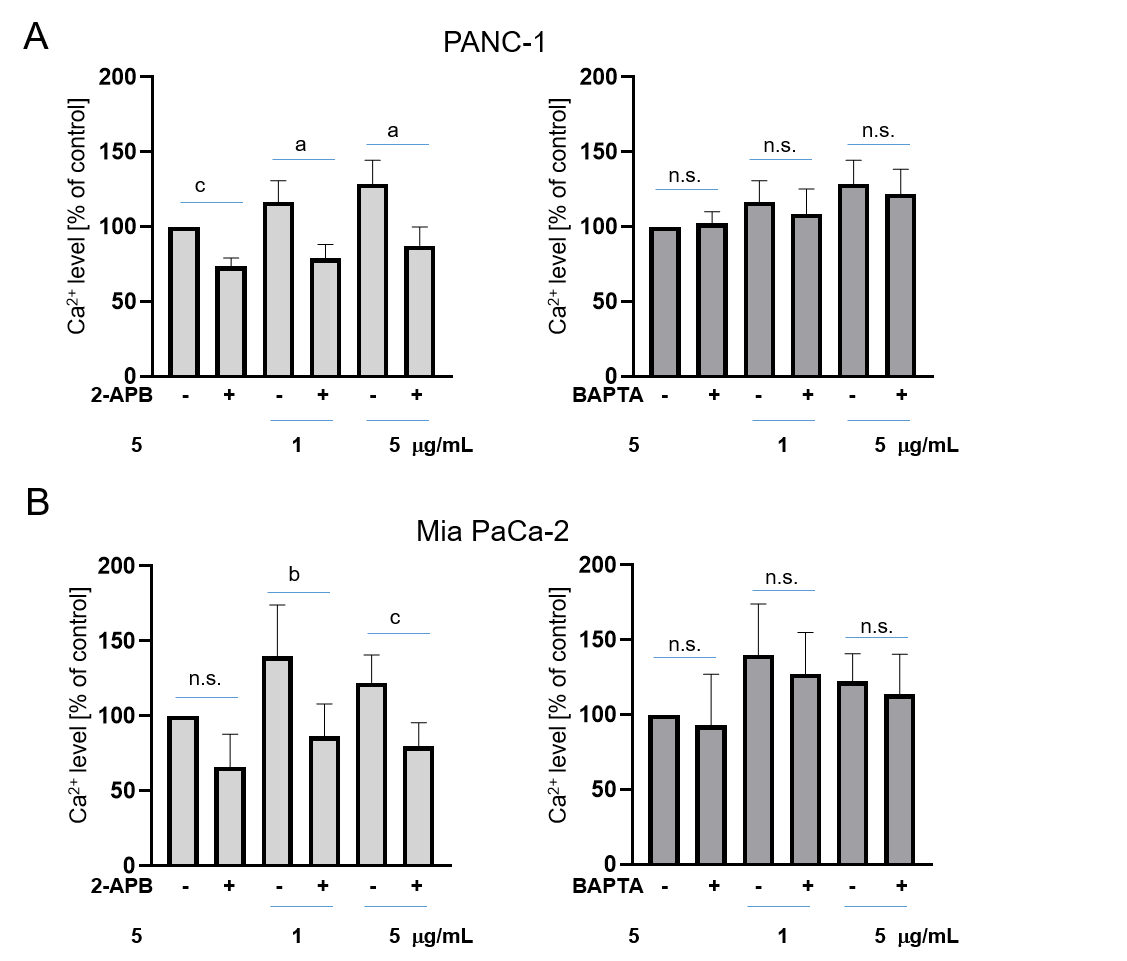
**

Elevation of cytosolic Ca^2+^ levels in pancreatic cancer cells results from its release from ER. The relative level of Ca^2+^ in PANC-1 (**A**) and Mia PaCa-2 (**B**) cells treated with vehicle (DMSO, 100%), **5** at indicated concentrations with or without inhibitor of IP3 receptors (2-APB at 30 μM, left panels) or extracellular calcium chelator (BAPTA, 10 μM, right panels) for 12 h. The data are shown as the mean ± SE (n=6-9). Statistical significance was determined by ANOVA followed by Sidak’s post-hoc tests: a - *P* < 0.0001, b - *P* < 0.001, c - *P* < 0.05, n.s. - not significant.
